# Gastric cancer: current and evolving treatment landscape

**DOI:** 10.1186/s40880-016-0147-6

**Published:** 2016-08-31

**Authors:** Weijing Sun, Li Yan

**Affiliations:** 1 University of Pittsburgh School of Medicine, Pittsburgh, PA 15232 USA; 2 US Chinese Anti-Cancer Association, Martinez, CA 94553 USA; 3 Beijing Cancer Hospital and Institute, Peking University School of Oncology, Beijing, 100142 P. R. China

**Keywords:** Gastric cancer, Treatment, Molecular medicine, Immunotherapy

## Abstract

Gastric (including gastroesophageal junction) cancer is the third leading cause of cancer-related death in the world. In China, an estimated 420,000 patients were diagnosed with gastric cancer in 2011, ranking this malignancy the second most prevalent cancer type and resulting in near 300,000 deaths. The treatment landscape of gastric cancer has evolved in recent years. Although systemic chemotherapy is still the mainstay treatment of metastatic disease, the introduction of agents targeting human epidermal growth factor receptor 2 and vascular endothelial growth factor/vascular endothelia growth factor receptor has brought this disease into the molecular and personalized medicine era. The preliminary yet encouraging clinical efficacy observed with immune checkpoint inhibitors, e.g., anti-programmed cell death protein 1/programmed death-ligand 1, will further shape the treatment landscape for gastric cancer. Molecular characterization of patients will play a critical role in developing new agents, as well as in implementing new treatment options for this disease.

## Background

Gastric (including gastroesophageal junction, GEJ) cancer (GC) is a very aggressive tumor and is the third leading cause of cancer-related deaths worldwide [1]. Near two-thirds of the gastric cancer patient population are found to have unresectable disease or distant metastases at the initial diagnosis in the Western world [2]. Even in those countries with screening programs established, up to 80% of patients who undergo a potential curative resection for gastric cancer develop locoregional or distant recurrence afterward [2]. In China, an estimated 420,000 patients were diagnosed with gastric cancer in 2011, ranking this malignancy the second most prevalent cancer type and resulting in near 300,000 deaths [3].

The treatment of patients with advanced gastric cancer remains a most challenging task in the clinical practice. Up to now, systemic chemotherapy alone remains to be the mainstay treatment for these patients [4]. Unfortunately, the disease often exhibits and develops resistance to chemotherapeutic agents with a relatively short period of disease and symptom control, and most patients would die within 1 year [5, 6]. Lack of understanding of gastric cancer at molecular and biological levels and the heterogeneity of the disease were two main reasons for having limited treatment choices and the poor outcome of the patients with advanced disease.

Recent significant advances in understanding the gastric cancer disease process from both biological and genomic prospective have brought target-oriented therapy for advanced gastric cancer into clinical research and practice. The cancer genome atlas (TCGA) project performed comprehensive molecular characterization of gastric adenocarcinoma and identified four major molecular subtypes as Epstein–Barr virus (EBV)-infected tumors, microsatellite instability (MSI) tumors, genomically stable tumors, and chromosomally unstable tumors [7]. Further genomic alterations and molecular subtypes/subclassifications may help guide the therapeutic implications for gastric cancer [8, 9]. We are going to outline the recent development of targeted treatments of gastric cancer and evolving landscapes for future research.

## Targeted agents

### Agents targeting HER2

Human epidermal growth factor receptor 2 (HER2, also known as ERBB2) is frequently amplified or overexpressed in up to 30% of advanced-stage gastric cancers. When combined with fluoropyrimidine/platinum chemotherapy, trastuzumab, a monoclonal antibody that targets HER2, extended median overall survival (OS) of patients with HER2-positive gastric or GEJ cancer from 11.1 months (95% confidence interval [CI] 10–13 months) to 13.8 months (95% CI 12–16 months) after the first-line treatment [10]. Trastuzumab in combination with chemotherapy subsequently became a new standard option for patients with HER2-positive advanced gastric or GEJ cancer. However, when lapatinib, a small-molecule tyrosine kinase inhibitor (TKI) targeting HER2 and epithelial growth factor receptor (EGFR), was combined with either first- (LOGiC) or second-line (TyTan) chemotherapies, no statistically significant improvement in OS was observed in gastric cancer patients with HER2 amplification defined by fluorescence in situ hybridization (FISH) [11]. One contributing factor might be the differences in patient selection criteria between trastuzumab and lapatinib trials. The subset of patients in the TyTAN trial who received lapatinib and whose tumors were HER2-positive as detected by FISH or strongly positive (+++) as detected by immunohistochemical (IHC) staining had prolonged OS (hazard ratio [HR] 0.59, *P* = 0.0176), highlighting the importance of assessing protein expression in addition to gene copy number in defining patients who are most likely to benefit from HER2-targeting agents [12].

Similar to the treatment option evolution in HER2-positive breast cancer, optimal synergistic combinations will likely be introduced to HER2-positive gastric cancer to further improve therapeutic efficacy. The phase III JACOB study tests trastuzumab/first-line chemotherapy with or without pertuzumab (an anti-HER2 antibody with a different binding domain than trastuzumab that inhibits HER2 dimerization with other HER family receptors). The phase II/III GATSBY study investigates the efficacy and safety of trastuzumab emtansine (an antibody–drug conjugate consisting of trastuzumab linked to the cytotoxic agent DM1) versus standard taxane treatment as second-line therapy. In addition, second-generation irreversible kinase inhibitors, such as afatinib, are currently being evaluated in a phase II study in trastuzumab-refractory HER2-positive GEJ cancer.

### Agents targeting VEGF/VEGFR-2

Vascular endothelial growth factor (VEGF)- and vascular endothelia growth factor receptor-2 (VEGFR-2)-mediated signaling and angiogenesis contribute to the pathogenesis of gastric cancer.

Bevacizumab, a VEGFA-directed monoclonal antibody, was tested in chemotherapy-naïve metastatic gastric (and GEJ) cancer patients in combination with chemotherapy. The combination of bevacizumab with chemotherapy was associated with significantly increased proportions of patients achieving an objective response and prolonged progression-free survival (PFS) as compared with chemotherapy alone; however, non-significantly prolonged OS was achieved [13]. The heterogeneity of the patient population (European/American versus Asian) and differences in the availability/options of second- and third-line therapy may affect the results. However, subsequent attempts failed to identify biomarkers that may help reveal subpopulation of patients who may benefit from bevacizumab [14].

Ramucirumab, a monoclonal antibody VEGFR-2 antagonist, demonstrated survival benefits as either a single agent (REGARD trial) [15] or in combination with paclitaxel (RAINBOW trial) [16] in patients whose disease progressed after prior first-line platinum-containing or fluoropyrimidine-containing chemotherapy. As a single agent, ramucirumab prolonged the median OS to 5.2 months in the treatment arm versus 3.8 months in the placebo arm (HR 0.776, 95% CI 0.603–0.998, *P* = 0.047). When combining ramucirumab with paclitaxel, the RAINBOW study showed that the median OS was 9·6 months (95% CI 8.5–10.8 months) in the combination arm and 7.4 months (95% CI 6.3–8.4 months) in paclitaxel plus placebo arm (HR 0.807, 95% CI 0.678–0.962, *P* = 0.017).

Apatinib, an oral small-molecule VEGFR-2 TKI, also significantly prolonged survival in patients with advanced gastric cancer who had progressed after first- or second-line chemotherapy. Median OS was significantly improved in the apatinib group compared with the placebo group (6.5 months; 95% CI, 4.8 to 7.6 v 4.7 months; 95% CI, 3.6 to 5.4; *P* = .0149; hazard ratio, 0.709; 95% CI, 0.537 to 0.937; *P* = .0156). Similarly, apatinib significantly prolonged median PFS compared with placebo (2.6 months; 95% CI, 2.0 to 2.9 v 1.8 months; 95% CI, 1.4 to 1.9; *P* < .001; hazard ratio, 0.444; 95% CI, 0.331 to 0.595; *P* < .001) [17].

### Agents targeting c-MET

Hepatocyte growth factor (HGF) and its receptor c-MET are implicated in cancer cell growth, invasion and metastasis, and angiogenesis. In gastric cancer, c-MET expression was reported in 26–74% of cases, and gene amplification in 2–23% of cases. Rilotumumab, a fully human monoclonal antibody that neutralizes HGF, showed some clinical activity in combination with epirubicin, cisplatin, and capecitabine as first-line treatment for gastric or GEJ adenocarcinoma in a phase II study [18]. However, this improvement in PFS was not translated into survival benefits in a confirmatory phase III study. In contrast, the RILOMET-1 phase III study results revealed that the rilotumumab and chemotherapy combination was statistically inferior compared with chemotherapy alone, with the median OS of 9.6 months (95% CI 7.9–11.4 months) versus 11.5 months (95% CI 9.7–13.1 months) (HR 1.37, 95% CI 1.06–1.78, *P* = 0.016) [19]. Similarly, onartuzumab, a monovalent anti-c-MET antibody, also failed to deliver clinical benefits when it was combined with mFOLFOX6 as the first-line treatment in patients with advanced gastric cancer [20]. Recent phase I study data of ABT-700, an anti-c-MET antibody, suggest that HGF/c-MET-directed treatment may require further patient selection [21]. Among 4 patients with *MET* gene amplification detected by FISH, 3 had partial response lasting 27, 18, and 24 weeks. On the other hand, 2 patients without *MET* amplification had progressive disease as the best response. *MET* amplification was only detected in metastatic/recurrent tumors but not the primary tumor tissue, suggesting that *MET* amplification might be more common in treatment-refractory tumors than in primary untreated tumors. These new insights may have potential to guide future development of HGF/c-MET-directed treatments.

## Immunotherapies

It is well known that tumors evade the host immune response via a multitude of mechanisms, therefore, develop resistance to immune effectors. These processes include, but not limit to, (1) expansion of immunosuppressive cells [regulatory T (T_reg_) cells and myeloid-derived suppressor cells (MDSCs)] in the tumor microenvironment; (2) elevation of various cytokines and chemokines [e.g., transforming growth factor-β (TGF-β), indoleamine 2,3-dioxygenase (IDO), interleukin (IL)-10]; and (3) co-inhibitory signaling pathways mediated via immune checkpoints [e.g., cytotoxic T-lymphocyte-associated protein-4 (CTLA-4), PD-1, T cell immunoglobulin- and mucin domain-containing molecule-3 (TIM-3), lymphocyte activation gene 3 (LAG3)] [22].

Immune checkpoint blockade strategy is now being actively evaluated in the management of gastrointestinal malignancies, including GEJ cancer. CTLA-4 is a key negative regulator of T-cell activation. It is constitutively expressed on the surface of Treg cells and inducibly expressed on activated T lymphocytes and monocytes. CTLA-4 up-regulation leads to reduced IL-2 production and IL-2 receptor expression as well as arrest of T cells at the G_1_ phase of the cell cycle. PD-1 is a co-inhibitory receptor expressed on the surface of activated T, B, and myeloid cells, and interacts with its ligands (PD-L1 and PD-L2) to prevent T-cell functioning. Antibody-mediated blockade of PD-1 or PD-L1 results in inhibition of this checkpoint, leading to T-cell functional activation and enhanced antitumor activity.

### Anti-CTLA-4

In a small phase II trial, tremelimumab, a fully humanized anti-CTLA-4 monoclonal antibody, was tested as a second-line therapy in 18 patients with gastric cancer [23]. Although the objective response rate was 5%, the median OS was 4.8 months and, therefore, similar to that expected with other chemotherapies in gastric cancer.

### Anti-PD-1 and anti-PD-L1

The single-agent activity of pembrolizumab, an anti-PD-1 antibody, was assessed in a phase I study KEYNOTE-012. The overall response rate (ORR) was 22% (95% CI 10%–39%) by central review and 33% (95% CI 19%–50%) by investigator review. The median time to response was 8 weeks (range 7–16 weeks), with a median response duration of 24 weeks (range 8+ to 33+ weeks). PD-L1 expression level was suggested being associated with ORR (one-sided *P* = 0.10). Median progression-free survival as assessed by central review was 1·9 months (95% CI 1.8–3.5), with 6-month progression-free survival of 26% (95% CI 13–41). Median overall survival was 11.4 months (95% CI 5.7–not reached). The proportion of patients alive at 6 months was 66% (95% CI 49–78) and at 12 months was 42% (95% CI 25–59). [24]. The other study of pemborlizumab for the heavily treated esophageal and GEJ carcinoma showed similar response with an ORR of 30.4%, a 6-month PFS rate of 30.4%, and a 12-month PFS rate of 21.7% [25]. Nivolumab, a fully human anti-PD-1 IgG4 monoclonal antibody, was tested in a phase I/II study (CheckMate-032) setting in patients with heavily treated metastatic gastric or GEJ cancer irrespective of PD-L1 status. The preliminary results were reported at the 2016 Gastrointestinal Cancers Symposium [26]. The study demonstrated that nivolumab monotherapy was well tolerated and demonstrated encouraging antitumor activity in heavily pretreated patients with gastric or GEJ cancer. Objective responses occurred in patients with either PD-L1-positive or -negative tumors. Similarly, avelumab, an anti-PD-1 IgG1 antibody, also demonstrated encouraging clinical activities in patients with advanced gastric cancer with an ORR of 15% and a 12-week PFS rate of 43.3% [27].

Based on these preliminary yet promising results, these anti-PD-1 antibodies (pembrolizumab, nivolumab, and avelumab) are now being investigated in various line settings of gastric cancer treatment either as a monotherapy or in combination with standard care chemotherapy with trial results available since 2017.

Furthermore, durvalumab, an anti-PD-L1 antibody, is being investigated in a phase Ib/II study in combination with tremelimumab in gastric cancer (https://clinicaltrials.gov/ct2/show/NCT02340975).

### Issues regarding clinic trail design, patient selection, study outcome assessment, and so on

Heterogeneity is a major huddle in clinical study design, data analysis, and application in the clinical practice for advanced gastric cancer: patients from Western versus Asian; different (intestinal versus diffuse) pathologic types; regimens of double-agent versus three-agent combination. The success of future drug development in gastric cancer will rely on the implementation of biomarker-driven patient selection to minimize the heterogeneity. Recent advancements in molecular characterization of gastric cancer have paved the way for genome-guided clinical trial and drug development. For example, in the EBV subgroup, amplification at 9p24.1 leads to up-regulation of PD-L1 and PD-L2, which in turn indicates a potential role of PD-1 axis blockade in treatment of these patients. The encouraging clinical activities observed in patients with mismatch repair deficiency support the exploration of anti-PD-1/PD-L1 approaches in patients with microsatellite instability (MSI) gastric cancer [28].

Practically, the traditional endpoints of the clinical trial and assessment method(s) may no longer fit the needs of advanced target-oriented studies. Currently, the OS rate is still used as the gold standard for the Food & Drug Administration (FDA) to approve new agents. However, it may not fairly represent the benefit of a particular tested agent because patients may receive various agents based on different targets after discontinuation from clinical trials. Functional imaging- or molecular characteristics-based measurement methods should be considered and developed for the future clinical study assessment.

## Conclusion

Gastric cancer research and treatment are undergoing a rapid transformation. With the advancements in molecular profiling and the introduction of targeted agents, gastric cancer is starting to enter the era of precision medicine. Immunotherapy will become a significant player albeit in a subset of patients. Global collaborative efforts are needed to further improve the treatment outcomes of gastric cancer.
